# Ethical considerations surrounding the response to Ebola: the Spanish experience

**DOI:** 10.1186/s12910-016-0135-z

**Published:** 2016-08-18

**Authors:** Miguel Ángel Royo-Bordonada, Fernando J. García López

**Affiliations:** 1 National School of Public Health, Institute of Health Carlos III, 8 Sinesio Delgado Street, Madrid, 28029 Spain; 2 National Centre for Epidemiology, Institute of Health Carlos III, Madrid, Spain

**Keywords:** Ebola, Ethics, Public health, Quarantine, Global justice, Informed consent

## Abstract

**Background:**

The recent Ebola virus disease (EVD) outbreak, with 28,646 reported cases and 11,323 deaths, was declared a public health emergency of international interest by the World Health Organisation. In Spain, a single reported case triggered a public health crisis of a markedly media-centred nature. The approach to the first EVD epidemic has given rise to various ethical considerations around the world. We address the most relevant ethical considerations emanating from the management of EVD in Spain.

**Main body:**

Firstly, for reasons of global justice and humanitarian assistance, rich countries have the duty to support poorer countries in building up their core public-health capacities. Secondly, quarantine for high-risk contacts might have been a disproportionate and not properly justified measure, which could have contributed to stigmatising contacts and spreading panic. Thirdly, when the first secondary case was reported in Spain, it is doubtful whether informed consent requirements were strictly complied with when disclosing information concerning the alleged accident potentially causing the contagion. Moreover, this information was used by the Regional Health Minister to blame the patient, evading his responsibility to ensure safe medical procedures for health workers. Finally, the patient received convalescent plasma for compassionate use from a colleague of the first missionary repatriated, who also participated in a research study in Spain, despite having previously been denied the chance of being transferred to Spain to receive treatment. This fact highlights the asymmetry in the relationship between rich and poor countries.

**Short conclusion:**

The management of this crisis highlighted the technical capacity of the health system and its professionals to respond effectively to public health emergencies caused by emerging diseases. This said, the failures in the protection of the EVD patient‘s privacy remind us that this aspect has to be borne in mind from the outset in crisis situations. Certain coercive measures, such as quarantine, should only be applied where there is some evidence that the benefit-risk balance could be favourable. Lastly, it is essential that research and interventions targeted at combating the fragility of the health systems in poor countries respond to reasons of global justice.

## Background

Compared to other endemic diseases in Africa that cause high morbidity and mortality, such as HIV/AIDS or diarrhoeic diseases [1], Ebola virus disease (EVD), with 28,646 reported cases and 11,323 deaths by 29 March 2016, when the World Health Organisation (WHO) Director-General declared the end of the Public Health Emergency of International Concern caused by the recent outbreak [2, 3], might be regarded as a small-scale killer. Nonetheless, the spread of the disease to a number of countries, its high lethality and the alarmist declarations concerning it, magnified by the news media, caused a state of panic which, in great measure, influenced the international response [4]. Spain, with a single reported case [5], in no way remained impervious to this phenomenon, which triggered a public health crisis of a markedly media-centred nature [6].

The approach to the largest outbreak of EVD in history has given rise to various ethical considerations, whether focusing on one specific aspect, such as the use of experimental treatments [7, 8], application of isolation measures [9], or obligations of developed countries in the fight against the epidemic [10], or viewed from a broader perspective [4, 11–13]. With the end of Ebola transmission in West Africa, this is a good time to review how situations with ethical implications were managed during the crisis, and draw lessons that will enable us to improve monitoring, preparedness and response capabilities vis-à-vis future public health emergencies [14]. This paper thus seeks to analyse the most relevant ethical considerations emanating from the management of EVD in Spain. Without any pretensions to achieving an exhaustive and complete account, we will address questions relating to the repatriation of aid workers with EVD, the application of quarantine to high-risk contacts, the dissemination of sensitive information about patients, and the use of experimental treatments.

## Discussion

### Repatriation of EVD sufferers

On 7 August 2014, a male missionary nurse who worked at a Monrovia hospital (Liberia) was repatriated to Spain to be treated for EVD at the hospital Carlos III in Madrid. In contrast, some of their work colleagues who belonged to their religious order and had also been affected had to remain in Liberia since they did not possess Spanish nationality. Whereas the Spanish missionary died on 12 August, a female colleague of his, who was denied to be transferred to Spain for treatment, was admitted to Elwa public hospital-a barrack-like building where patients were crowded together without the necessary therapeutic and hygienic conditions-managed to survive the disease and on 25 August walked out of the “death camp”, as it was called by the locals [15]. On 21 September, a missionary physician was repatriated to Spain from Sierra Leone, in order to be treated for EVD at the hospital Carlos III, where he died 4 days later.

The repatriation of missionaries responds to our obligation, as a society, to provide the best care available to aid workers who, through supererogatory actions in the name of solidarity, are willing to place their lives at risk in order to help others [16]. Repatriation also benefits the rest of the patients, by maximising the probability that their caregivers will survive and minimising the risk of dissuading other professionals who might otherwise be considering the possibility of travelling to the area to help with their care [17]. Spanish law recognises the right of aid workers to repatriation in the event of accident or severe disease. To cover the cost of repatriation, humanitarian aid agencies must previously take out an insurance policy that covers the expenses of their workers [18]. In the apparent absence of the necessary insurance policy in the case of the Ebola-stricken missionaries who were transferred to Spain, it was the state that paid the costs of their repatriation, waiving the right to have these reimbursed by the religious order [19].

The repatriation of missionaries raises the question of the different treatment accorded to Spanish aid workers and the African health workers, who were on the front line and were the principal victims of infection [10]. It seems clear that the duty of attending to health workers who risk their lives falls to their respective countries of origin. However, given the enormous shortages faced by such states and the profound world-wide inequality in the distribution of resources, even those most leery of the blessings of international aid acknowledge the existence of global interdependence (social, economic, physical and moral) [20] and the obligations of rich countries to poorer countries. These obligations should offset the injustices that prevent people from leading a minimally decent life regardless of their place of birth, an example of this being the existence of fragile health systems which have been the determinants of the spread of the current Ebola outbreak [10]. In addition to the reasons of global justice, most people recognise that individuals and societies have an obligation to provide humanitarian assistance to others when the cost of this burden is minimal [10]. In the mid and long term, this amounts to helping to strengthen these countries‘ health systems but, in the midst of the crisis, the priority lies in providing the necessary health-care personnel, training and technical means to combat the epidemic, while reinforcing patient treatment capabilities and the control of transmission chain [21]. Indeed, this is what the WHO Member States agreed upon in the International Health Regulations some 10 years ago, by undertaking to support developing countries in building up their core public-health capacities [22]. Countries which had an important presence and a certain logistic infrastructure in the area were the first to take steps in this direction [23]. The case of Cuba, a country without interests in the region, which sent a sizeable contingent of health professionals to the affected area, is particularly laudable [24]. Two independent groups of experts have set up similar recommendations to counter the threat of infectious diseases crisis in the future [25, 26]. The global community should set a strategy to develop countries’ public-health capacities by promoting investment by local governments, providing technical and funding support when needed, and ensuring external assessment of national capacities. In addition, WHO should create a Centre for Health Emergency Preparedness and Response, put aside a contingency fund, establish effective mechanisms for coordination, escalation and cooperation with regional networks and non-state actors in health crisis, and help to enable and accelerate research.

Other controversial aspects of the repatriation were the financial cost of the operation and the risk of introducing an infectious agent into a country that was free of the disease and had negligible experience in its management [27, 28]. Furthermore, doubts were raised as to whether the level of suitability of infrastructures and training of professionals in Spain was optimal for handling this type of patient [29]. Specifically, the hospital unit equipped to deal with infectious emergencies was in the process of being dismantled and its reopening had to be quickly improvised in order to care for the repatriated patients. Hence, one has to ask oneself about possible alternative actions that might have guaranteed the missionaries a level of care equivalent to what they would have received in Spain but left them in West Africa, such as dispatching a health-care team with the necessary means to set up a field hospital, something which was indeed proposed by some public health experts at the time and from which many other patients might well have benefited [30]. The financial cost of such an aid action on the ground can be very variable [31] but is estimated to exceed 3 million euros [32], a figure higher than the total cost of repatriation and treatment in Europe, which is also very variable but is nevertheless put at slightly over one million euros [33]. Even so, this is a minimal sum for a high-income country such as ours, which, just a few weeks after a number of public health experts had called on European governments to request urgent aid to control the epidemic in Africa [34], allocated 7 million euros to funding prevention projects in EVD outbreak-affected countries and their border areas [35]. This sum is approximately half the budget allocated at the time to meeting the needs arising from the EVD outbreak in Spain, without taking into account the resources allocated by regional authorities to preparing the staff and facilities of countless hospitals to treat Ebola patients, when the vast majority of these will never get to see a single case [36]. Following repatriation of Spanish personnel who had become infected, Spain contributed additional funds through the European Union and sent a group of epidemiologists to the affected area.

Lastly, the use of exceptional protection measures during repatriation —disproportionate in relation to the risk of transmission of the virus—and their dissemination by the mass media were questioned by some experts, who warned of the risk of transmitting a distorted perception of the risk and thereby contributing to the population’s panic [37].

### Quarantining of high-risk contacts

On October 6, 2014, a secondary case was reported in Spain, caused by the first known human-to-human transmission of Ebola virus disease outside Africa [5]. The patient was an assistant nurse who had cared for the two missionaries repatriated from West Africa. Although symptom-onset occurred on 29 September and the patient telephoned the prevention unit of the hospital where she worked on the following day, she was not diagnosed until 1 week later. The ultimate reasons for this delay are not entirely clear, but were seemingly related in the initial stages to a “wait-and-see” attitude to vague symptoms, with fever that failed to reach the level established in the protocol for case-definition purposes [5, 38, 39]. The patient subsequently went to her primary care centre on her own initiative but failed to report her previous contact with Ebola patients and was prescribed paracetamol [40]. Over the course of the following days, there was a repetition of the telephone contacts between the patient and the Madrid Health Service but it was not until 6 October, after the symptoms had become exacerbated, that she was finally admitted to the hospital [41]. Experiences like this have shown that, in a sporadic case context, diagnostic criteria ought to be applied with greater flexibility than in an epidemic context, with the protocols for investigating possible cases having been amended to enhance their sensitivity when it comes to detecting Ebola patients [39].

Disproportionate and sensationalist mass media coverage plus ineffective communication during the first days after the assistant nurse had tested positive contributed to widespread panic [6]. After some initial confusion, during which the health authorities transmitted the sensation that they were incapable of controlling the situation created by the infection of the female health worker, the our government reacted in a co-ordinated manner in response to the surveillance system’s slowness to detect the contagion and the ensuing alarm caused by the situation. On the one hand, it set up a national Ebola management committee [42], which immediately implemented a comprehensive and transparent communication campaign, one of the first measures recommended by the experts to reduce confusion [16]; and on the other, it implemented a new version of the EVD- protocol, with substantial changes in the contact monitoring and surveillance procedure [39, 43]. The modifications to the protocol included the following, seemingly reasonable, measures: changing the clinical criteria used to define cases under investigation to enhance diagnostic sensitivity; providing instructions for evaluating the fever on an individualised basis; notifying primary and specialised care centres of any persons subject to surveillance by reason of their status as Ebola case contacts; and requiring all such contacts to report any travel outside their region of residence, during which time they would be expected to remain permanently reachable by telephone, and in the event of needing medical attention, to inform their monitoring officer prior to seeking care. Furthermore, quarantine for high-risk contacts was established on the grounds of avoiding any risk of diagnostic delay -such as that which occurred in the case of the infected assistant nurse- and facilitating rapid isolation. One would nevertheless have to ask whether quarantine was a reasonable, proportional measure. Although the scientific evidence shows that asymptomatic persons are not contagious [44] and that the risk of spread is low during the early febrile phase of illness [45], it may nonetheless be reasonable to adopt a more aggressive attitude to this marginal risk in the context of sporadic cases than in that of an epidemic. Based on this same evidence, however, the previous version of the Spanish protocol noted that there was no transmissibility in the incubation period and made no provision for the possibility of quarantine for high risk contacts [39]. This was precisely the line of reasoning pursued by a District Court to reject an application by the governor of Maine (USA) for quarantine to be imposed on a female nurse who had returned from Sierra Leone, where she had gone with *Médecins Sans Frontières* to help combat the EVD outbreak [46]. Firstly, in addition to restricting individual rights, quarantine can contribute to stigmatising contacts, inducing some to conceal their condition, and to undermining health professionals’ willingness to treat patients, which would result in a diminished capacity to combat the epidemic [47, 48]. Although we do not know this with certainty, the introduction of quarantine would possibly contribute to the stigmatisation of contacts and their families observed in Spain [41]. Secondly, quarantine diverts resources which could be used to combat in more effective ways the epidemic or attend to other urgent health needs [49]. In Spain, high-risk contacts were given the choice of home or hospital quarantine and all of them opted for hospital quarantine, citing among other reasons the fear of infecting family members, in spite of the fact that, apparently, no appropriate justification for quarantine was given to them [50]. Extraordinary measures such as quarantine unsupported by scientific evidence could perhaps be justified during a crisis, if these served to control the emergency by preventing the spread of panic. Yet one would equally need to have some kind of evidence to show the capacity of quarantine to generate trust in the population or when it comes to applying the precautionary principle [51]. However, the issue of contradictory messages by the health authorities would be expected to give rise to confusion and mistrust in the population: by repeatedly laying stress on the fact that the disease is exclusively transmitted by direct contact with patients who already present with symptoms of the disease and yet at the same time placing asymptomatic contacts in quarantine, one is giving to understand that the latter also pose a risk of contagion to the remainder of the population. The fact that all the high-risk contacts voluntarily opted for hospital-based quarantine, including health professionals, suggests that this measure failed to improve the population’s perception of risk. Our experience during EVD patient-management training courses, held at the request of the special Ebola Management Committee at the National School of Public Health from 15 October 2014, with the aim of reinforcing the training of bio-health professionals [52], indicated that an altered perception of the risk persisted among some professionals, as a result of the panic situation created, an impression that was shared by other professionals involved in the management of the crisis [6]. Lastly, though there was a delay in the diagnosis of the case, attributable to a surveillance system failure, the remaining measures listed above appeared to be sufficient to remedy the shortfalls detected without any need to resort to quarantine. A country such as Spain, with an almost universal, largely state-run health system of acknowledged quality [53], is able to put in place those measures albeit with one serious drawback, ie, that immigrants without residence permits have been practically excluded from the health system since 2012 [54]. Furthermore, despite the fact that, legally speaking, exclusion from the health system would in no way affect high-risk public health situations, many immigrants have nevertheless assumed that they are totally excluded. Consequently, such exclusion, aside from being enormously unjust, poses evident difficulties for epidemiological and public health surveillance of potentially imported diseases like EVD. Quarantine decisions must be based on science and not on panic or hysteria [55]. In public health crisis, with distorted public perceptions of risk and lack of evidence regarding which method can be more effective to control the situation, governments can appeal to the precautionary principle. However, before opting for extreme coercive measures, such as quarantine, alternative courses of action can be explored using the principle of least restrictive or coercive means, like offering quarantine on a voluntary basis versus monitoring fever and other clinical symptoms through daily at home visits by health professionals. Whatever the case, a public and transparent justification of a very cautious approach, with measures that go beyond what would be strictly needed in terms of potential risk, would prevent population and health professionals from mistrusting health authorities for contradictory messages. Otherwise, one could be contributing to create a state of opinion not altogether averse to restrictions being placed, without the necessary justification, on personal freedom in crisis situations. The case of the Aliens Internment Centre in Aluche, a Madrid neighbourhood, where a group of immigrants was totally unjustifiably and arbitrarily kept in isolation for 18 h without receiving sustenance of any kind, should serve as a warning of the risk of allowing oneself to be swayed by panic when it comes to taking decisions in emergency situations [56].

### Dissemination of sensitive information

The EVD episode in Spain has highlighted the need to pay more attention to the mechanisms required to ensure patient privacy in crisis situations. The name of the patient affected was made public from the very start [57]. However, some persons whose participation in the crisis aroused media interest, such as the primary care physician who attended to the patient days before she was diagnosed, managed to keep their identity private. This shows that the privacy of patients and health professionals affected by a public health crisis can be ensured without necessarily curtailing the right to information of public interest.

Two days after making the patient’s name public, the medical team at the EVD-reference hospital in Madrid, acting with the patient’s express consent, publicly revealed information concerning the alleged accident that could have caused the contagion. According to these statements, made at the hospital entrance a short while after having talked to the patient various times and asking for her consent to release the details, the patient had acknowledged that she might have touched her face with the gloves when she was removing the personal protective equipment (PPE) which she had donned to attend to the second of the repatriated health workers. Subsequently, the patient issued other statements in which she did not admit to this accident and, on being asked again by her medical team, said that she felt confused about the matter [58]. This incident raises at least two questions:

1. Was the patient’s consent to disclose the information regarding the possible accident that might have caused contagion an autonomous decision? In order for informed consent to be considered valid, three conditions must be met [59]: (a) the patient must have sufficient information about the consequences of the decision to be taken; (b) the patient must be able to understand the relevant information and decide in accordance with his/her preferences; and, (c) the decision must be taken voluntarily, in the absence of internal and external coercion. Bearing in mind the sheer magnitude of the media circus that surrounded the case, the social alarm and panic generated, and the patient’s clinical condition (ie, she had been diagnosed with a highly lethal disease), it is doubtful whether these requirements were strictly complied with. However preserved the patient’s ability to comprehend, possibly neither the patient nor the medical team who attended to her could have foreseen the consequences of disclosing the above information. Furthermore, the coercion stemming from the enormous pressure exerted by the media and the patient’s personal and professional circle, highlighted by her contradictory statements, raises doubts about the degree to which her decision was voluntary.
2. What was the purpose of publicly disclosing the above information so hastily? At that particular point in time, identifying a possibility of infection was of great importance to all professionals who were attending Ebola patients, since it reduced suspicion of the PPE’s inability to afford protection. What is not so clear is whether this information was relevant to the general public. In the context of confusion and social alarm, it could be understood as a measure intended to combat panic, and transmit trust and confidence to the population, showing that the source of the contagion had been discovered and that the situation was under control. Nevertheless, far from helping to allay panic, the statements instead served to spark off a media and political scramble in search of a culprit to blame for what had occurred, thereby doing irreparable harm to the investigation targeted at clarifying what had happened [60]. The Madrid Regional Health Minister used this revelation to blame the patient publicly for what had occurred, accusing her via television of lying, concealing information and incompetence, with derogatory comments such as, “One doesn”t need a Master’s degree to understand how to put on and take off a suit but some people are undoubtedly quicker to learn than others” [61]. Some communication media sympathetic to the government fanned the fire, by blaming and stigmatising the victim, accusing her of being imprudent and putting many people at risk, in a strategy that appeared to be designed to deflect the blame and shield the health authorities [62]. If the alleged accident that might have caused the contagion did indeed take place during the removal of the PPE, then this must have happened as a result of unintentional carelessness by the patient within an overall context of flaws in the protective procedures and means available, especially a lack of an adequate supervision, which are ultimately to blame for the alleged failure, a point repeatedly made by some of the health professionals at the hospital [63]. In neither of these two cases can blame or responsibility be attached to the patient for the accident or its consequences. What is in fact called for here is to implement systems that are designed to prevent errors and, in a worst case scenario, are able to detect and correct them before they cause any harm [64]. Indeed, if the PPE-removal protocol had been appropriate, the accident should have been detected at the time it happened. The focus must be on system errors rather than personal errors [65]. However, the regional government, in charge of direct health care of Ebola patients, rather than admitting its responsibility for not preventing the contagion blamed and shamed the patient for it, without any compensation for the damage inflicted. It is true that the patient was to blame for her failure to disclose to the primary care centre that she had been in contact with infected people. But, even so, an active surveillance system of high-risk contacts at that time should have prevented this forgetfulness from happening.

The final invasion of the patient’s privacy took place when a number of news media published a Reuters News Agency photograph taken with a telephoto zoom lens, showing the assistant nurse in her hospital room [66]. Some media afterwards withdrew the photograph, including *El País*, one of Spain’s leading mass-circulation dailies, which publicly acknowledged that the image should not have been published and issued an apology to its readers via a “tweet” [67]. Although some legal and other experts invoke the right to information on the grounds of the public interest in the case, the majority opinion was that the photograph should never have been published, since it was breach of the patient’s right to privacy and image (personality) rights [68].

## Use of experimental treatments

The first repatriated patient received ZMapp. The patient who was a secondary case received two experimental treatments, namely, convalescent plasma collected from two EVD survivors and high-dose favipiravir [40]. Although these patients gave their informed consent to receiving these experimental treatments, they were for compassionate use, which meant that they were not within the framework of any clinical trial. Such a compassionate use had not been previously evaluated by a research ethics committee. Nevertheless, the pertinent research ethics committee did discuss the application of treatments for future potential patients. In defence of the clinicians who were tasked with caring for the patient, it is very difficult to take the stance of positioning the experimental treatments within the framework of a clinical trial in a situation where there is only one patient with the disease and it is practically impossible to organise a multinational clinical trial within the space of a few days. Evaluation by a clinical research ethics committee, advisable in compassionate use but not required by the Spanish law, would certainly have been more feasible, despite the rapidity required to formulate the possible benefits and risks of experimental treatments and then discuss the matter at a committee meeting. During the secondary case’s clinical disease course, she presented with an acute respiratory distress syndrome which met the diagnostic criteria for possible transfusion-related acute lung injury, after receiving five units of convalescent plasma from two different EVD survivors, though the possibility that this might have been secondary to EVD cannot be ruled out [40].

The case of the Spanish assistant nurse raises an additional question, since one of the donors of the plasma received was a work colleague and member of same religious order as the first missionary repatriated, who had managed to survive in Liberia after she had been denied the chance of being transferred to Spain to receive treatment. Yet, once she had recovered from the disease, when her serum could potentially serve to treat other patients, she was transferred to Spain [69] and not only donated her serum, but also participated in a study to investigate what the best time would be for extracting the plasma from convalescent patients [70]. Regardless of the source of the funding for transferring the female missionary, these facts highlight an asymmetry in the relationship between rich and poor countries, which could contribute to generating mistrust and opposition to future research initiatives in these countries, due to fear of possible exploitation of the affected parties. The USA government has invested considerable money in recent years in treatments and vaccines for Ebola, seeing it as a threat to its national security [71]. Even so, in order to eradicate the terrible social inequalities that bar many persons from aspiring to a minimally decent life, it is essential that research and interventions targeted at combating the fragility of the health systems in poor countries respond to reasons of global justice.

## Conclusions

In crisis situations, the speed in response coupled with the ability to adapt swiftly to changing situations are some of the keys to success. In the case before us, despite the initial confusion, associated with failures in communication, the immediate implementation of a centralised, comprehensive and transparent communication campaign, with spokespersons being chosen in accordance with their technical and scientific profiles, helped restore part of the population’s lost confidence. This would appear to indicate that something has been learnt from communication failures in previous crises, such as that which marked mad cow disease [72]. The continuous updating of the Ebola patient management protocol, by adapting and improving it with the aid of contributions made by experts from different disciplines in line with newly received data and experience gained in the management of patients and contacts, is yet another indicator of the system’s organisational capacity to respond swiftly and effectively to new, rapidly changing challenges through co-ordinated actions.

This said, the management of this crisis has also highlighted some areas for improvement, particularly those relating to the ethical considerations inherent in some of the public health measures applied. The serious failures that occurred in the protection of the EVD patient’s privacy are a good example of this, while the fact that private information about other persons involved in the crisis was not divulged shows that it is possible to protect the confidentiality of affected parties in crisis situations if this aspect is borne in mind from the outset. Certain measures not supported by scientific evidence, such as the quarantining of EVD high-risk contacts, should only be applied, publicly and transparently, on the basis of the precautionary principle, where there is some evidence that the benefit-risk balance could be favourable. But this was not so clear in this particular case, where similar level of protection of population and health professionals could have been reached with less restrictive measures, preventing from the risk of distrust of health authorities caused by contradictory messages. Failure to do so entails the risk of contributing to create a state of opinion favourably disposed towards restrictions being placed on personal freedoms without any real justification in crisis situations.

Lastly, the fragility of poor countries’ health systems has been pinpointed as being one of the principal limitations of the global capacity to respond to international public health emergencies [14]. To date, rich countries’ response to such shortcomings and the need to conduct research into treatments and vaccines for endemic diseases in poor countries has essentially been the product of self-interest, for legitimate reasons of global security, which is primarily aimed at protecting the citizens of these countries against threats arising beyond their frontiers. However, the only way to put an end to the enormous social inequalities that underlie this fragility and achieve a sustained, meaningful improvement in global health and the international response capacity to public health emergencies, lies in tackling the above-mentioned challenges from the standpoint of solidarity and global justice. Regarding Spain, where the epidemic will likely never break out, part of the funds wasted preparing the staff and facilities of countless hospitals to treat Ebola patients would have better been used to help combat the epidemic in Africa. Unlike other countries, such as Cuba or the UK, the Spanish government did not encourage the staff of the National Health System to volunteer in stopping the outbreak in Africa. To help in countering the threat of infectious-diseases crisis and reducing avoidable suffering in the future, Spain should join the global community efforts to develop countries’ public-health capacities, providing technical and funding support to poor countries when needed and supporting the WHO’s leadership role in this challenge.

## Abbreviations

AIDS: Acquired Immunodeficiency Syndrome
EVD: Ebola Virus Disease
HIV: Human Immunodeficiency Virus
PPE: Personal Protective Equipment
WHO: World Health Organisation
